# Dianilinedibromidozinc(II)

**DOI:** 10.1107/S1600536809043694

**Published:** 2009-10-28

**Authors:** Ejaz, Onur Sahin, Islam Ullah Khan

**Affiliations:** a Materials Chemistry Laboratry, Department of Chemistry, GC University, Lahore 54000, Pakistan; b Department of Physics, Ondokuz Mayis University, TR-55139 Samsun, Turkey

## Abstract

In the title compound, [ZnBr_2_(C_6_H_7_N)_2_], the Zn atom (site symmetry 2) adopts a distorted tetra­hedral ZnN_2_Br_2_ geometry. In the crystal, mol­ecules are linked by N—H⋯Br hydrogen bonds, generating sheets containing *R*_2_^2^(8) loops.

## Related literature

For background to the applications of zinc complexes, see: Ibrahim *et al.* (2003 ▶); Nesterova *et al.* (2005 ▶); Park *et al.* (2008 ▶); Wu *et al.* (2008 ▶). For graph-set theory, see: Bernstein *et al.* (1995 ▶).
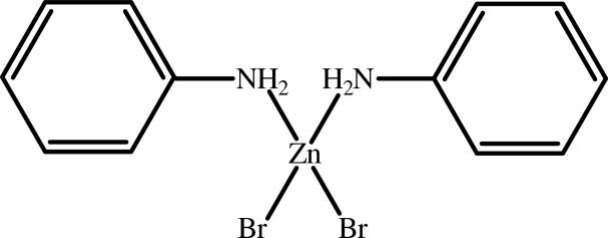


## Experimental

### 

#### Crystal data

[ZnBr_2_(C_6_H_7_N)_2_]
*M* *_r_* = 411.44
Monoclinic, 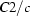

*a* = 25.7545 (16) Å
*b* = 4.9415 (3) Å
*c* = 12.1919 (8) Å
β = 111.035 (3)°
*V* = 1448.21 (16) Å^3^
*Z* = 4
Mo *K*α radiation
μ = 7.19 mm^−1^
*T* = 296 K
0.43 × 0.41 × 0.40 mm

#### Data collection

Bruker APEXII CCD diffractometer
Absorption correction: none
7092 measured reflections
1796 independent reflections
1489 reflections with *I* > 2σ(*I*)
*R*_int_ = 0.026

#### Refinement

*R*[*F* ^2^ > 2σ(*F* ^2^)] = 0.024
*wR*(*F* ^2^) = 0.068
*S* = 1.18
1796 reflections
86 parameters
H atoms treated by a mixture of independent and constrained refinement
Δρ_max_ = 0.36 e Å^−3^
Δρ_min_ = −0.60 e Å^−3^

### 

Data collection: *APEX2* (Bruker, 2007 ▶); cell refinement: *SAINT* (Bruker, 2007 ▶); data reduction: *SAINT*; program(s) used to solve structure: *SHELXS97* (Sheldrick, 2008 ▶); program(s) used to refine structure: *SHELXL97* (Sheldrick, 2008 ▶); molecular graphics: *ORTEP-3* (Farrugia, 1997 ▶); software used to prepare material for publication: *WinGX* (Farrugia, 1999 ▶).

## Supplementary Material

Crystal structure: contains datablocks I, global. DOI: 10.1107/S1600536809043694/hb5146sup1.cif

Structure factors: contains datablocks I. DOI: 10.1107/S1600536809043694/hb5146Isup2.hkl

Additional supplementary materials:  crystallographic information; 3D view; checkCIF report

## Figures and Tables

**Table 1:** Selected bond lengths (Å)

Zn1—N1	2.057 (2)
Zn1—Br1	2.3851 (3)

**Table 2:** Hydrogen-bond geometry (Å, °)

*D*—H⋯*A*	*D*—H	H⋯*A*	*D*⋯*A*	*D*—H⋯*A*
N1—H1*A*⋯Br1^i^	0.90 (3)	2.75 (3)	3.597 (3)	157 (2)
N1—H2*A*⋯Br1^ii^	0.87 (3)	2.76 (3)	3.564 (3)	156 (3)

Symmetry codes: (i) 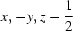; (ii) 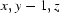.

**Fig. 1.**
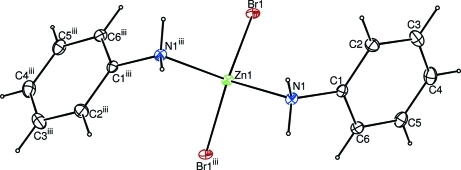
The molecular structure of (I), showing displacement ellipsoids drawn at the 30% probability level. [Symmety code: (iii) 1 - x, y, 3/2 - z.]

**Fig. 2.**
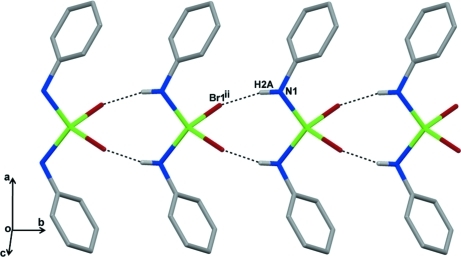
Part of the crystal structure of the title compound, showing the formation of an R22(8) dimer along [010].

**Fig. 3.**
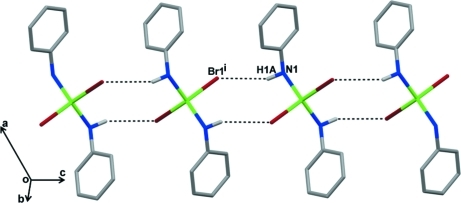
Part of the crystal structure of the title compound, showing the formation of an R22(8) dimer along [001]. Hydrogen bonds are indicated by dashed lines. H atoms not involved in these interactions have been omitted for clarity. (Symmetry codes as in Table 1.)

## supplementary crystallographic information

### Comment

Researches have worked on synthesis and X-ray studies of organo-zinc complexes for their applications in catalysis (Ibrahim *et al.*, 2003, Park *et al.*, 2008) and supramolecular chemistry (Nesterova *et al.*, 2005). These complexes act as fluorescent probe for labeling proteins (Wu *et al.*, 2008). Herein, we report the synthesis and crystal structure of the title compound, (I).

The molecular structure of (I) is presented in Fig. 1. The compound crystallizes in the space group *C*2/*c* with *Z*' = 1/2. The Zn^II^ ion is located on a 2-fold axis and is coordinated by two Br atoms [Zn1—Br/Br1^iii^ = 2.3851 (3) Å] and two amino N atoms from aniline ligands [Zn1—N1/N1^iii^ = 2.057 (2) Å] [symmetry code: (iii) 1 - *x*, *y*, 3/2 - *z*]. The geometry around the Zn^II^ ion is that of a tetrahedron.  The benzene ring plane is approximately planar, with maximum deviation from the least-squares plane being 0.004 (2)Å for atom C2.

Molecules of the title compound are linked in to shetts by a combination of N—H···Br hydrogen bonds (Table 1). Amino atom N1 in the reference molecule at (*x*, *y*, *z*) acts as hydrogen-bond donor, *via* H2A, respectively, to atom Br1 in the molecule at (*x*, *y* - 1, *z*), so forming a C(4)[*R*_2_^2^(8)] (Bernstein *et al.*, 1995) chain of rings running parallel to the [010] direction (Fig. 2). Similarly, amino atom N1 in the reference molecule at (*x*, *y*, *z*) acts as hydrogen-bond donor, *via* H1A, respectively, to atom Br1 in the molecule at (*x*, -*y*, *z* - 1/2), so forming a C(4)[*R*_2_^2^(8)] chain of rings running parallel to the [001] direction and centrosymmetric *R*_2_^2^(8) ring centred at (1/2, 0, 1/2) (Fig. 3).

### Experimental

Zinc bromide (1.125 g, 5 mmol) was added to distilled water (20 ml). Aniline (0.93 g, 10 mmol) was added to the above solution and stirred at room temperature for 5 minutes. White precipitate formed was filtered off, washed with distilled water, dried and recrystallized in methanol to yield colourless blocks of (I).

### Refinement

All C-bonded H atoms were refined using a riding model, with C—H distances constrained to 0.93Å and with *U*_iso_ = 1.2*U*_eq_(C). Amino H atoms were located in difference map and refined freely.

### Crystal data

**Table:**

[ZnBr_2_(C_6_H_7_N)_2_]	*Z* = 4
*M**_r_* = 411.44	*F*(000) = 800
Monoclinic, *C*2/*c*	*D*_x_ = 1.887 Mg m^−^^3^
Hall symbol: -C 2yc	Mo *K*α radiation, λ = 0.71073 Å
*a* = 25.7545 (16) Å	Cell parameters from 7092 reflections
*b* = 4.9415 (3) Å	µ = 7.19 mm^−^^1^
*c* = 12.1919 (8) Å	*T* = 296 K
β = 111.035 (3)°	Block, colourless
*V* = 1448.21 (16) Å^3^	0.43 × 0.41 × 0.40 mm

### Data collection

**Table:**

Bruker APEXII CCD diffractometer	1489 reflections with *I* > 2σ(*I*)
Radiation source: fine-focus sealed tube	*R*_int_ = 0.026
graphite	θ_max_ = 28.3°, θ_min_ = 1.7°
φ and ω scans	*h* = −34→32
7092 measured reflections	*k* = −4→6
1796 independent reflections	*l* = −16→16

### Refinement

**Table:**

Refinement on *F*^2^	Primary atom site location: structure-invariant direct methods
Least-squares matrix: full	Secondary atom site location: difference Fourier map
*R*[*F*^2^ > 2σ(*F*^2^)] = 0.024	Hydrogen site location: inferred from neighbouring sites
*wR*(*F*^2^) = 0.068	H atoms treated by a mixture of independent and constrained refinement
*S* = 1.18	*w* = 1/[σ^2^(*F*_o_^2^) + (0.035*P*)^2^] where *P* = (*F*_o_^2^ + 2*F*_c_^2^)/3
1796 reflections	(Δ/σ)_max_ = 0.001
86 parameters	Δρ_max_ = 0.36 e Å^−^^3^
0 restraints	Δρ_min_ = −0.60 e Å^−^^3^

### Special details

**Table:**

Geometry. All e.s.d.'s (except the e.s.d. in the dihedral angle between two l.s. planes) are estimated using the full covariance matrix. The cell e.s.d.'s are taken into account individually in the estimation of e.s.d.'s in distances, angles and torsion angles; correlations between e.s.d.'s in cell parameters are only used when they are defined by crystal symmetry. An approximate (isotropic) treatment of cell e.s.d.'s is used for estimating e.s.d.'s involving l.s. planes.
Refinement. Refinement of *F*^2^ against ALL reflections. The weighted *R*-factor *wR* and goodness of fit *S* are based on *F*^2^, conventional *R*-factors *R* are based on *F*, with *F* set to zero for negative *F*^2^. The threshold expression of *F*^2^ > σ(*F*^2^) is used only for calculating *R*-factors(gt) *etc*. and is not relevant to the choice of reflections for refinement. *R*-factors based on *F*^2^ are statistically about twice as large as those based on *F*, and *R*- factors based on ALL data will be even larger.

### Fractional atomic coordinates and isotropic or equivalent isotropic displacement parameters (Å^2^)

**Table:**

	*x*	*y*	*z*	*U*_iso_*/*U*_eq_	
C1	0.61172 (10)	−0.0453 (4)	0.7448 (2)	0.0332 (5)	
C2	0.65438 (13)	−0.1002 (6)	0.8489 (3)	0.0483 (7)	
H2	0.6498	−0.2303	0.8998	0.058*	
C3	0.70386 (14)	0.0379 (7)	0.8775 (3)	0.0618 (8)	
H3	0.7328	−0.0013	0.9475	0.074*	
C4	0.71102 (14)	0.2333 (7)	0.8038 (3)	0.0620 (9)	
H4	0.7445	0.3269	0.8240	0.074*	
C5	0.66823 (14)	0.2887 (6)	0.7000 (3)	0.0543 (8)	
H5	0.6728	0.4200	0.6495	0.065*	
C6	0.61856 (12)	0.1502 (5)	0.6703 (2)	0.0428 (6)	
H6	0.5897	0.1887	0.6001	0.051*	
N1	0.55851 (9)	−0.1809 (4)	0.7147 (2)	0.0350 (5)	
H1A	0.5450 (13)	−0.231 (6)	0.638 (3)	0.052 (8)*	
H2A	0.5592 (14)	−0.334 (6)	0.750 (3)	0.058 (9)*	
Zn1	0.5000	0.05076 (7)	0.7500	0.03217 (12)	
Br1	0.546312 (11)	0.32589 (5)	0.91739 (2)	0.04053 (11)	

### Atomic displacement parameters (Å^2^)

**Table:**

	*U*^11^	*U*^22^	*U*^33^	*U*^12^	*U*^13^	*U*^23^
C1	0.0370 (14)	0.0280 (12)	0.0384 (13)	0.0019 (10)	0.0181 (11)	−0.0068 (9)
C2	0.0499 (18)	0.0449 (14)	0.0472 (16)	0.0039 (14)	0.0136 (14)	0.0063 (13)
C3	0.0452 (19)	0.064 (2)	0.063 (2)	0.0048 (16)	0.0033 (16)	−0.0045 (16)
C4	0.048 (2)	0.0555 (18)	0.085 (3)	−0.0135 (15)	0.0271 (19)	−0.0186 (18)
C5	0.057 (2)	0.0478 (16)	0.069 (2)	−0.0092 (14)	0.0348 (18)	−0.0008 (14)
C6	0.0466 (17)	0.0427 (15)	0.0412 (15)	−0.0038 (12)	0.0184 (13)	−0.0023 (11)
N1	0.0406 (13)	0.0297 (11)	0.0374 (12)	−0.0024 (9)	0.0176 (10)	−0.0032 (9)
Zn1	0.0379 (2)	0.0308 (2)	0.0310 (2)	0.000	0.01626 (18)	0.000
Br1	0.0558 (2)	0.03761 (16)	0.02807 (15)	−0.00087 (11)	0.01487 (12)	−0.00366 (9)

### Geometric parameters (Å, °)

**Table:**

C1—C2	1.375 (4)	C5—C6	1.380 (4)
C1—C6	1.380 (3)	C5—H5	0.9300
C1—N1	1.450 (3)	C6—H6	0.9300
C2—C3	1.376 (4)	N1—H1A	0.90 (3)
C2—H2	0.9300	N1—H2A	0.87 (3)
C3—C4	1.376 (5)	Zn1—N1	2.057 (2)
C3—H3	0.9300	Zn1—N1^i^	2.057 (2)
C4—C5	1.375 (5)	Zn1—Br1	2.3851 (3)
C4—H4	0.9300	Zn1—Br1^i^	2.3851 (3)
			
C2—C1—C6	119.8 (2)	C5—C6—C1	120.0 (3)
C2—C1—N1	120.8 (2)	C5—C6—H6	120.0
C6—C1—N1	119.3 (2)	C1—C6—H6	120.0
C1—C2—C3	119.8 (3)	C1—N1—Zn1	112.76 (14)
C1—C2—H2	120.1	C1—N1—H1A	111.5 (19)
C3—C2—H2	120.1	Zn1—N1—H1A	109 (2)
C2—C3—C4	120.8 (3)	C1—N1—H2A	115 (2)
C2—C3—H3	119.6	Zn1—N1—H2A	106 (2)
C4—C3—H3	119.6	H1A—N1—H2A	102 (3)
C5—C4—C3	119.4 (3)	N1^i^—Zn1—N1	112.35 (13)
C5—C4—H4	120.3	N1^i^—Zn1—Br1	108.50 (7)
C3—C4—H4	120.3	N1—Zn1—Br1	108.50 (7)
C4—C5—C6	120.3 (3)	N1^i^—Zn1—Br1^i^	108.50 (7)
C4—C5—H5	119.9	N1—Zn1—Br1^i^	108.50 (7)
C6—C5—H5	119.9	Br1—Zn1—Br1^i^	110.49 (5)
			
C6—C1—C2—C3	−0.8 (4)	N1—C1—C6—C5	177.4 (2)
N1—C1—C2—C3	−177.7 (2)	C2—C1—N1—Zn1	98.8 (2)
C1—C2—C3—C4	0.8 (5)	C6—C1—N1—Zn1	−78.1 (2)
C2—C3—C4—C5	−0.5 (5)	C1—N1—Zn1—N1^i^	−152.2 (2)
C3—C4—C5—C6	0.2 (5)	C1—N1—Zn1—Br1	−32.26 (19)
C4—C5—C6—C1	−0.2 (4)	C1—N1—Zn1—Br1^i^	87.82 (17)
C2—C1—C6—C5	0.5 (4)		

Symmetry codes: (i) −*x*+1, *y*, −*z*+3/2.

### Hydrogen-bond geometry (Å, °)

**Table:**

*D*—H···*A*	*D*—H	H···*A*	*D*···*A*	*D*—H···*A*
N1—H1A···Br1^ii^	0.90 (3)	2.75 (3)	3.597 (3)	157 (2)
N1—H2A···Br1^iii^	0.87 (3)	2.76 (3)	3.564 (3)	156 (3)

Symmetry codes: (ii) *x*, −*y*, *z*−1/2; (iii) *x*, *y*−1, *z*.
